# Altered microglial response to Aβ plaques in APPPS1-21 mice heterozygous for TREM2

**DOI:** 10.1186/1750-1326-9-20

**Published:** 2014-06-03

**Authors:** Jason D Ulrich, Mary Beth Finn, Yaming Wang, Alice Shen, Thomas E Mahan, Hong Jiang, Floy R Stewart, Laura Piccio, Marco Colonna, David M Holtzman

**Affiliations:** 1 Department of Neurology, Washington University School of Medicine, Saint Louis, Missouri 63110, USA; 2 Department of Pathology and Immunology, Washington University School of Medicine, Saint Louis, Missouri 63110, USA; 3 Department of Medicine, Washington University School of Medicine, Saint Louis, Missouri 63110, USA; 4 Developmental Biology, Washington University School of Medicine, Saint Louis, Missouri 63110, USA; 5 Hope Center for Neurological Disorders, Washington University School of Medicine, Saint Louis, Missouri 63110, USA; 6 Knight Alzheimer’s Disease Research Center, Washington University School of Medicine, Saint Louis, Missouri 63110, USA

**Keywords:** Alzheimer’s disease, TREM2, Microglia, Amyloid β

## Abstract

**Background:**

Recent genome-wide association studies linked variants in *TREM2* to a strong increase in the odds of developing Alzheimer’s disease. The mechanism by which TREM2 influences the susceptibility to Alzheimer’s disease is currently unknown. TREM2 is expressed by microglia and is thought to regulate phagocytic and inflammatory microglial responses to brain pathology. Given that a single allele of variant *TREM2*, likely resulting in a loss of function, conferred an increased risk of developing Alzheimer’s disease, we tested whether loss of one functional *trem2* allele would affect Aβ plaque deposition or the microglial response to Aβ pathology in APPPS1-21 mice.

**Results:**

There was no significant difference in Aβ deposition in 3-month old or 7-month old APPPS1-21 mice expressing one or two copies of *trem2*. However, 3-month old mice with one copy of *trem2* exhibited a marked decrease in the number and size of plaque-associated microglia. While there were no statistically significant differences in cytokine levels or markers of microglial activation in 3- or 7-month old animals, there were trends towards decreased expression of NOS2, C1qa, and IL1a in 3-month old TREM2^+/−^ vs. TREM2^+/+^ mice.

**Conclusions:**

Loss of a single copy of *trem2* had no effect on Aβ pathology, but altered the morphological phenotype of plaque-associated microglia. These data suggest that TREM2 is important for the microglial response to Aβ deposition but that a 50% decrease inTREM2 expression does not affect Aβ plaque burden.

## Background

One of the hallmarks of Alzheimer’s disease (AD) is the extracellular deposition of amyloid-β (Aβ) peptide in the brain parenchyma as amyloid plaques. Autosomal dominant Alzheimer’s disease (ADAD) is an early-onset form of AD which is caused by rare mutations in amyloid β (A4) precursor protein (*APP*), presenilin-1 (*PSEN1*), or presenilin-2 (*PSEN2*) that alter Aβ production [1]. Genetic variants also influence the risk of developing the more common late onset form of AD (LOAD). To date the two strongest identified LOAD genetic risk factors are the well-studied apolipoprotein ϵ4 (*APOE4*) allele and several recently identified variants in the triggering receptor expressed on myeloid cells-2 (*TREM2*) gene [2-4]. While these variants are not common, since *TREM2* variants strongly increase the risk of developing AD, understanding how TREM2 dysfunction affects AD pathology could yield novel therapeutic strategies.

*TREM2* encodes a transmembrane protein possessing an extracellular IgG-like ligand binding domain and an intracellular region that associates with the immunoreceptor tyrosine based activating motif (ITAM)-containing signaling adaptor protein DAP12 [5]. Individuals that are homozygous for loss of function mutations in either *TREM2* or *TYROBP* (DAP12) suffer from polycystic lipomembranous osteodysplasia and sclerosing leukoencephalopathy (PLOSL) which is characterized by early onset dementia and cystic bone lesions [6]. Within the brain, TREM2 is expressed by microglia and appears to regulate microglial-mediated phagocytic clearance of cellular debris and the inflammatory response of microglia to pathology, however the endogenous ligand(s) for TREM2 are unknown [7-10]. TREM2 expression is increased in plaque-associated microglia in APP23 and TgCRND8 mice suggesting that TREM2 is involved in the microglial response to Aβ plaque deposition [3,11,12]. The role of microglia in AD is complex and incompletely understood. Microglia rapidly migrate to Aβ plaque deposits and acquire an amoeboid “activated” morphology [13,14]. Pro-inflammatory M1-like microglial activation is generally considered neurotoxic, while pro-phagocytic M2-like activation can lead to microglial clearance of Aβ in murine AD models [15]. Since TREM2 is implicated in regulating the phagocytic and inflammatory function of macrophages, TREM2 dysfunction could conceivably increase Aβ plaque burden through decreased phagocytic clearance of Aβ and/or promote a neurotoxic, inflammatory microglial phenotype in response to Aβ deposition.

In this study we tested whether loss of a single *trem2* allele affected Aβ plaque burden in APPPS1-21 mice[16]. To facilitate analysis of microglia we took advantage of the CX3CR1-GFP mice which in the CNS express GFP specifically within microglia [17]. Although we did not observe a significant difference in Aβ plaque deposition between TREM2^+/+^ and TREM2^+/−^ mice, there was a substantial decrease in plaque-associated microglia in TREM2^+/−^ mice compared to TREM2^+/+^ mice. These data suggest that TREM2 function may affect the microglial response to Aβ pathology.

## Results

### TREM2 hemizygosity does not affect Aβ deposition in 3-month old APPPS1-21 mice

**Figure 1:**
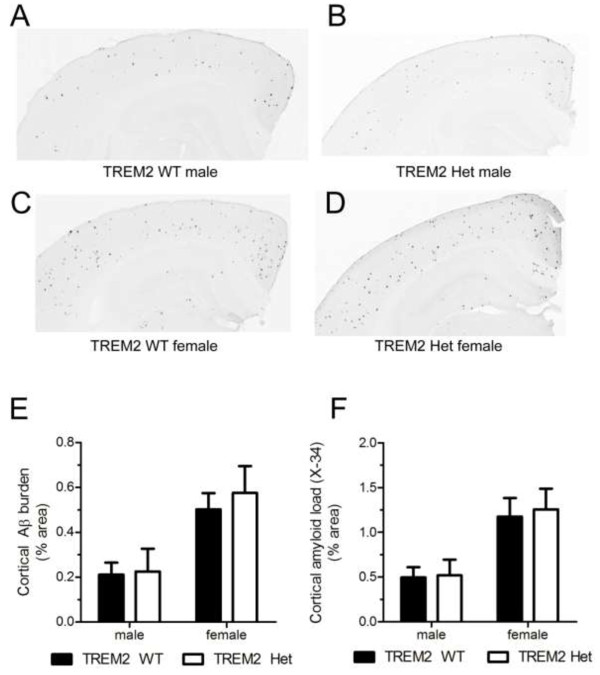
**TREM2 heterozygosity does not affect Aβ plaque deposition in 3-month old APPPS1-21 mice. (A-D)** Representative coronal brain sections from 3-month old from male TREM2 WT **(A)**, male TREM2 Het **(B)**, female TREM2 WT **(C)** and female TREM2 Het **(D)**. Sections were immunostained with a biotinylated anti-Aβ antibody HJ3.4. **(E)** Quantification of the percentage of cortical area occupied by Aβ immunostaining. Two-way ANOVA analysis found a significant effect of gender (F_1,39_ = 13.63, p = 0.0007), but not genotype (F_1,39_ = 0.25, p = 0.62). **(F)** Quantification of the percentage of cortical area occupied by X-34 staining. Two-way ANOVA analysis found a significant effect of gender (F_1,39_ = 14.33, p = 0.0005), but not genotype (F_1,39_ = 0.08, p = 0.78; TREM2 WT (male, n = 12; female, n = 12) TREM2 Het (male, n = 9; female, n = 10)). Data are presented as mean ± SEM.

**Figure 2:**
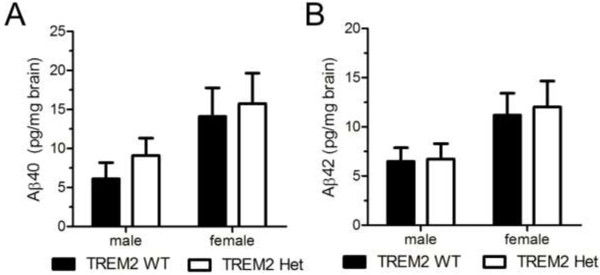
**TREM2 heterozygosity does not affect PBS-insoluble Aβ levels in 3-month old APPPS1-21 mice. (A)** Mean concentrations of PBS-insoluble Aβ_40_ in cortical tissue from TREM2 WT (male, n = 13; female, n = 12) and TREM2 Het (male, n = 8; female n = 10) mice were determined by ELISA. Two-way ANOVA analysis found a significant effect of gender (F_1,38_ = 5.49, p = 0.02), but not genotype (F_1,38_ = 0.55, p = 0.46). **(B)** Mean concentrations of PBS-insoluble Aβ_42_ in cortical tissue from TREM2 WT (male, n = 13; female, n = 12) and TREM2 Het (male, n = 8; female n = 10) mice were determined by ELISA. Two-way ANOVA analysis found a significant effect of gender (F_1,38_ = 5.96, p = 0.02), but not genotype (F_1,38_ = 0.07, p = 0.79). Data are presented as mean ± SEM.

Individuals that are heterozygous for *TREM2* variants predicted to result in a decrease or loss of TREM2 function in the affected allele, have increased odds of developing AD [3,4]. TREM2 expression in microglia is associated with phagocytic clearance of extracellular debris, such as apoptotic neurons, raising the possibility that TREM2 could regulate microglial mediated clearance of extracellular Aβ, and ultimately amyloid plaque deposition [8]. We compared the amount of cortical Aβ deposition in the early stages of plaque formation using 3-month old APPPS1-21 mice expressing two copies (TREM2^+/+^, CX3CR1^+/GFP^, APPPS1-21, referred to as TREM2 WT) (Figure 1A,C) or one copy of TREM2 (TREM2^+/−^, CX3CR1^+/GFP^, APPPS1-21, referred to as TREM2 Het) (Figure 1B,D). We observed significantly more Aβ deposition in female mice compared to male mice for both TREM2 WT and TREM2 Het mice; however, we did not detect a significant effect of TREM2 copy number on Aβ deposition (Figure 1E). We further examined whether TREM2 affected amyloid deposition by staining brain sections with X-34, a dye that binds to fibrillar Aβ [18]. Again, we observed approximately double the amount of amyloid staining in female mice compared to male mice, but no significant difference between TREM2 WT and TREM2 Het mice (Figure 1F). We also biochemically assessed Aβ accumulation by measuring the level of PBS insoluble Aβ_40_ and Aβ_42_ from TREM2 WT and TREM2 Het cortical tissue. As expected given the immunohistological data, female mice had significantly higher amounts of insoluble Aβ_40_ and Aβ_42_ than male mice. However, there was no genotype-dependent difference in the levels of insoluble Aβ_40_ or Aβ_42_ (Figure 2). Taken together, these data suggest that TREM2 hemizygosity has no effect on Aβ plaque burden during the early stages of Aβ deposition.

### Altered microglial response to Aβ plaque deposits in TREM2 Het mice

**Figure 3:**
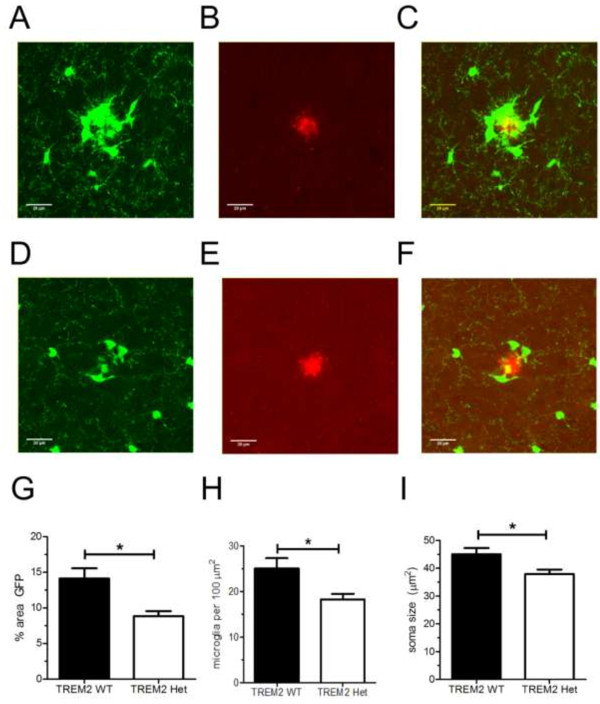
**Decreased plaque-associated microglia in TREM2 Het mice. (A-F)** Representative images of Alexa568-HJ3.4-stained plaque and GFP-expressing microglia from TREM2 WT **(A-C)** and TREM2 Het **(D-F)** mice. **(G)** The number of microglia per 100 μm^2^ within a 20 μm^2^ radius of an Aβ plaque in TREM2 WT mice (25.1 ± 2.25, n = 10) and TREM2 Het mice (18.3 ± 1.24, n = 9) was compared using a Mann–Whitney test (p = 0.03). **(H)** The mean soma size off plaque-associated microglia in TREM2 WT mice (45.0 ± 2.23 μm^2^, n = 10) and TREM2 Het mice (37.9 ± 1.57 μm^2^, n = 9) was compared using a Mann–Whitney test (p = 0.03). **(I)** The percent area covered by plaque-associated microglia in TREM2 WT mice (14.1 ± 1.4%, n = 10) and TREM2 Het mice (8.8 ± 0.71%, n = 9) was compared using a Mann–Whitney test (p = 0.01).

Microglia migrate to sites of plaque deposition and acquire an activated state that may restrict plaque growth or produce a neurotoxic inflammatory response [13,14]. We hypothesized that TREM2 could regulate the microglial localization around amyloid plaques. To test this hypothesis we compared the percentage of area covered by GFP-expressing microglia within a 20 μm radius of the edge of Aβ plaques in 3-month old TREM2 WT and TREM2 Het mice. Since female mice exhibited more robust Aβ deposition than male mice, we chose to analyze the microglial response in female mice. TREM2 Het mice exhibited a decrease in the density of plaque-associated microglia compared to TREM2 WT mice (Figure 3G). Microglial soma were also smaller in TREM2 Het compared to TREM2 WT mice (Figure 3H). The overall effect of the reduced number and size of plaque-associated microglia in TREM2 Het mice was very strong; there was a ~40% reduction in microglial coverage around Aβ plaques in TREM2 Het mice compared to TREM2 WT mice (Figure 3I). Thus, although TREM2 WT and TREM2 Het mice exhibited similar levels of Aβ deposition, there was a significant decrease in microglial localization near Aβ plaques.

### No significant alterations in gene expression or cytokine levels in 3-month old TREM2 Het mice

**Figure 4:**
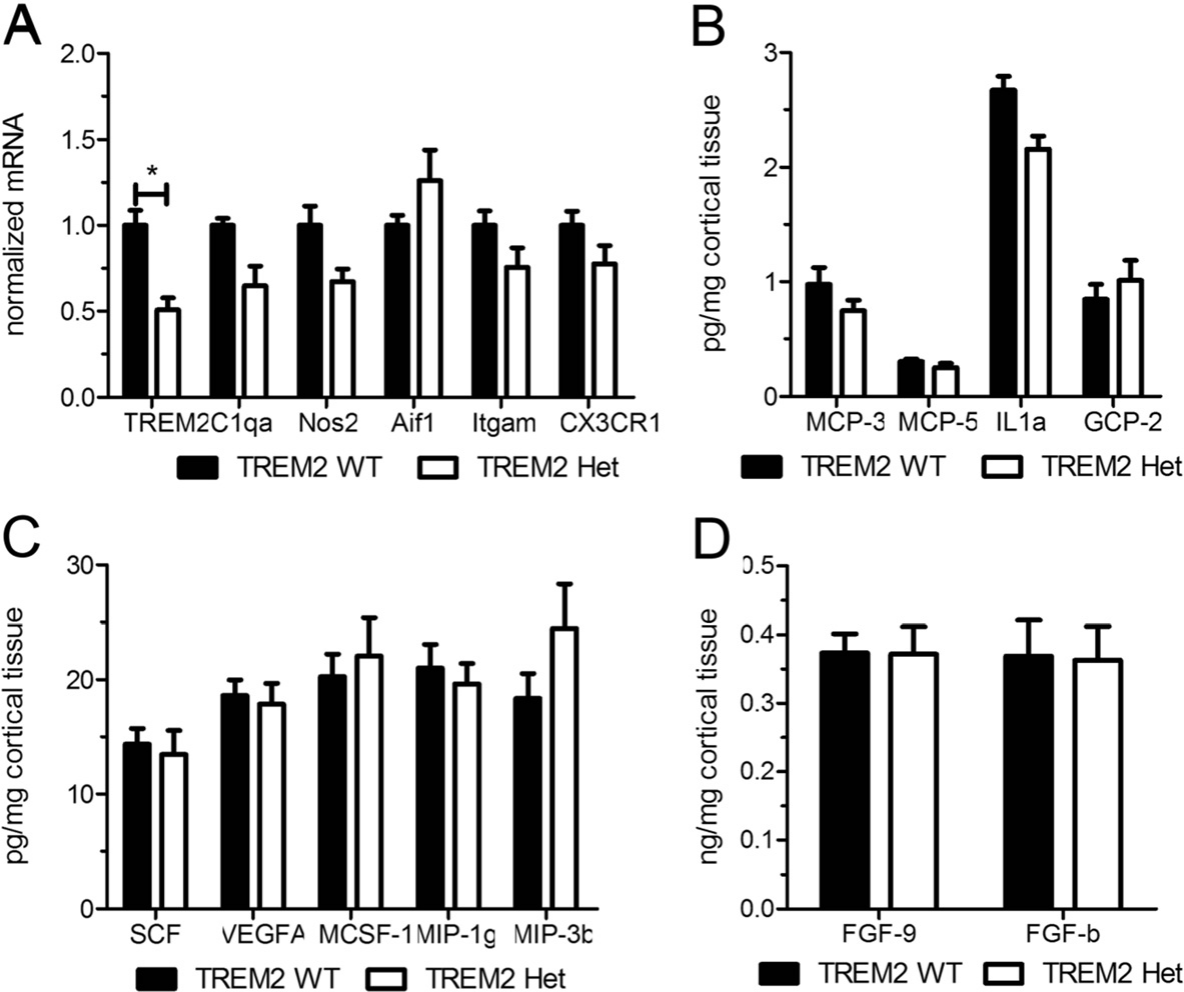
**TREM2 heterozygosity does not significantly affect the inflammatory milieu in 3-month old APPPS1-21 mice. (A)** Quantification of relative gene expression of microglial markers and NOS2 in TREM2 WT and TREM2 Het mice (n = 4-6 mice/genotype). For each mRNA analyzed TREM2 Het values were normalized and compared to TREM2 WT values using a t-test followed by a Benjamini-Hochberg p-value correction for multiple comparisons. **(B-D)** Levels of inflammatory cytokines in cortical tissue from TREM2 WT (n = 6) and TREM2 Het (n = 6) mice were compared using a t-test followed by Benjamini-Hochberg p-value correction for multiple comparisons. Cytokine levels are plotted on different axis for clarity of presentation. All data are presented as mean ± SEM, *corrected p < 0.05.

Given the decreased localization of microglia to Aβ plaques and altered morphology of plaque-associated microglia, we next tested whether there were alterations in the expression levels of microglial markers associated with AD, or in the inflammatory milieu of the brain, of TREM2 Het compared to TREM2 WT mice. We first compared the mRNA levels in cortical tissue of the microglial markers TREM2, C1qa, Aif1, Itgam (CD11b/CR3), and CX3CR1. Predictably, the relative level of TREM2 mRNA in TREM2 Het mice was ~50% that of TREM2 WT mice (Figure 4A). The expression levels of the other microglial markers were not significantly different following correction for multiple comparisons. Interestingly though, there was a trend towards an approximately 35% decrease in C1qa levels in TREM2 Het compared to TREM2 WT mice (corrected p-value 0.091) (Figure 4A). We also compared the expression level of NOS2 which can be upregulated during pro-inflammatory microglial responses [19]. Although not statistically significant, there was a 33% decrease in NOS2 expression in TREM2 Het mice as compared to WT (corrected p-value 0.090) (Figure 4A). To more broadly characterize the effects of reduced TREM2 expression on the inflammatory milieu of the brain in response to Aβ pathology we measured the levels of cytokines from cortical tissue lysates of TREM2 WT and TREM2 Het mice (Figure 4B-D). Many inflammatory cytokines, such as TNFα and IL1β, fell below the limit of detection, possibly due to the early stage of Aβ pathology detected in 3-month old APPPS1-21 mice. While we did not detect any significant differences in cytokine levels between TREM2 WT and TREM2 Het mice, there was a trend towards lower levels of the pro-inflammatory cytokine IL1α in TREM2 Het (24.0 pg/mL ± 1.3, n = 6) compared to TREM2 WT (29.7 pg/mL ± 1.3, n = 6) mice (corrected p-value 0.12) (Figure 4B). Overall, the qRT-PCR and cytokine data suggest a trend towards lower inflammation in TREM2 Het compared to TREM2 WT mice, consistent with the decreased microglial localization to Aβ plaque deposits in TREM2 Het mice.

### No difference in Aβ deposition or microglial activation marker expression in 7-month old TREM2 WT and TREM2 Het mice

**Figure 5:**
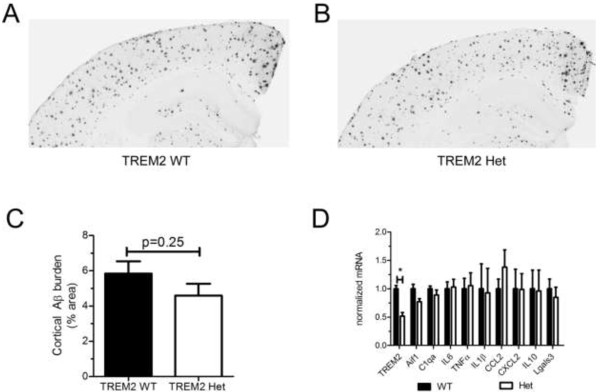
**TREM2 hemizygosity does not significantly affect Aβ plaque burden or expression of M1/M2 microglial markers in 7-month old APPPS1-21 mice. (A and B)** Representative coronal brain sections from 7-month old female TREM2 WT **(A)** and TREM2 Het **(B)** mice. Sections were immunostained with the biotinylated anti-Aβ antibody, HJ3.4. **(C)** Quantification of the cortical area occupied by Aβ immunostaining. TREM2 WT (5.85 ± 0.69%, n = 9) and TREM2 Het (4.60 ± 0.66%, n = 7) were statistically compared using a Mann Whitney test (p = 0.25). **(D)** Quantification of microglial mRNA expression in TREM2 WT and TREM2 Het mice. For each mRNA examined TREM2 Het were normalized and compared to TREM2 WT mice using a t-test followed by Benamini-Hochberg p-value correction for multiple comparisons. All data are presented as mean ± SEM (n = 3-7 mice per group), *p < 0.05.

We next assessed whether TREM2 hemizygosity would alter Aβ plaque burden in more advanced stages of pathology by comparing the level of cortical Aβ plaque deposition in 7-month old TREM2 WT and TREM2 Het mice. At 7-months of age both TREM2 WT and TREM2 Het mice exhibited robust cortical Aβ plaque deposition (Figure 5A-B). However, as in 3-month old mice, there was no significant difference in the level of cortical Aβ plaque between TREM2 WT and TREM2 Het mice (Figure 5C).To determine if there were differences in the microglial activation state at a more advanced stage of Aβ pathology we isolated microglia from 7-month old TREM2 WT and TREM2 Het mice and performed qRT-PCR to measure the expression level of genes associated with M1 or M2 polarization. As expected we observed a ~50% reduction in TREM2 mRNA levels in TREM2 Het mice (Figure 5D). However, we observed no difference in the expression level of M1 markers IL1β, IL6, TNFα, CCL2, or CXCL2, or in the level of M2 markers IL10 and Lgals3 (Figure 5D). We also observed no statistically significant difference in the expression level of C1qa or Aif1 between 7-month old TREM2 WT and TREM2 Het mice. These data indicate that there is no effect of TREM2 hemizygosity on Aβ plaque deposition or microglial gene expression that we assessed during later stages of Aβ pathology.

## Discussion

*TREM2* variants, particularly the R47H mutation, strongly increase the risk of developing AD, however how TREM2 affects AD and AD pathology is unknown [3,4]. Here, we report a decrease in the number and size of plaque-associated microglia in 3-month old TREM2 Het mice as compared to TREM2 WT mice, suggesting that TREM2 regulates the microglial response to Aβ plaque deposition. To the best of our knowledge this is the first report of an observable microglial phenotype in hemizygous TREM2 mice. As the resident macrophages in the brain, microglia are hypothesized to mediate both a beneficial phagocytic clearance of Aβ from the brain, and a detrimental chronic inflammatory phenotype resulting in neurotoxicity [20]. Longitudinal *in vivo* imaging studies demonstrate that microglia rapidly form clusters around Aβ deposits, although the molecular determinants of microglial migration to Aβ deposits are poorly understood [13,14]. Plaque-associated microglia also assume an amoeboid morphology with larger cell somas than non-plaque associated microglia [21]. Our data indicates that plaque-associated microglia in TREM2 Het mice are smaller than in TREM2 WT mice, which may indicate a defect in microglial activation.

We observed a reduced number of plaque-associated microglia in TREM2 Het mice compared to TREM2 WT mice. The reduced microglial response in TREM2 Het mice could result from defective microglial activation, migration, survival, or proliferation. Genetic network analysis of TREM2 expressed in the brain linked TREM2 to genes involved in regulating cytoskeletal rearrangements required for phagocytosis and migration [22]. In the periphery TREM2-DAP12 signaling is important for chemotaxic macrophage migration to the lungs following exposure to cigarette smoke, supporting the hypothesis that TREM2 can regulate macrophage migration to sites of injury [23]. Microglial proliferation also contributes to the population of plaque associated microglia [21,24]. TREM2 regulates macrophage-colony stimulating factor (M-CSF)-induced osteoclast precursor cell proliferation [25]. Microglia express colony-stimulating factor 1 receptor (CSF1R) which is regulates both microglial proliferation and viability [26-28]. Therefore, one potential explanation is that TREM2 is important for CSF1R-dependent responses to pathology. Further studies will be needed to characterize the mechanistic basis for how TREM2 regulates the number of plaque-associated microglia.

Despite the reduction in plaque-associated microglia, we did not observe any statistically significant difference in the expression of inflammatory cytokines or genes associated with microglial activation in TREM2 Het and TREM2 WT mice in either 3-month or 7-month old animals. TREM2-DAP12 signaling inhibits Toll-like receptor (TLR)-dependent cytokine production and bone marrow derived macrophages from TREM2 KO mice exhibit increased expressed inflammatory cytokine production in response to microbial stimulation [29]. Similarly, knockdown of TREM2 expression in microglia co-cultured with apoptotic neurons resulted in increased production of TNFα and NOS2 [8]. In contrast, TREM2 KO mice exhibited decreased inflammatory cytokine production compared to TREM2 WT mice in the middle cerebral artery occlusion model of stroke concomitant with decreased localization of activated microglia within the glial scar [10]. Thus the overall effect of TREM2 dysfunction on inflammatory signaling may depend upon the precise pathological context. It is also important to note that the effects of TREM2 on cytokine production were described in the context of a complete loss of TREM2 function, such as occurs in PLOSL. TREM2 Het mice may retain sufficient TREM2 function to properly regulate cytokine production. One caveat to our study is that although we did not detect a compensatory upregulation of TREM2 at the mRNA level, we were unable to quantify TREM2 protein expression in brain lysate by western blot using currently available reagents. Therefore, we cannot exclude the possibility that TREM2 protein expression is post-transcriptionally modified to compensate for loss of TREM2 expression.

Although we did not detect a TREM2-dependent difference in Aβ plaque burden, another microglial-associated protein genetically associated with AD, CD33, appears to substantially influence Aβ deposition [30-33]. CD33 appears to inhibit microglial uptake of Aβ *in vitro* and genetic deletion of *CD33* in APP_SWE_/PS1_ΔE9_ mice reduces Aβ plaque burden [30]. Furthermore, individuals possessing *CD33* variants that were associated with increased odds of developing AD exhibited higher CD33 expression and protective *CD33* variants resulted in lower CD33 expression [30,34]. Taken together, the effects of CD33 on microglial clearance of Aβ and the TREM2-dependent effects on plaque-associated microglia reported in this study, suggest that alterations in microglial function may impact different stages of AD pathogenesis.

Although we observed a strong decrease in microglial localization near Aβ plaques at 3 months, we did not observe a significant difference in Aβ plaque burden between TREM2 WT and TREM2 Het mice at either 3 or 7 months. One hypothesized function of plaque-associated microglia is to restrict the growth of Aβ plaque, which would imply that a decrease in plaque-associated microglia could result in larger Aβ plaques [14]. However, a previous study demonstrated that a four-week ablation of microglia had no effect on Aβ plaque burden in APPPS1-21 or APP23 mice, suggesting that, over the short term, Aβ plaque growth was not significantly impacted by microglia [35]. TREM2 is thought to promote microglial phagocytic activity, and therefore decreased functional TREM2 expression could result in reduced clearance of Aβ and a subsequent increase in plaque deposition [8]. Although in this study we did not test the phagocytic function of TREM2, the lack of significant effect of TREM2 hemizygosity on Aβ plaque burden does not support the hypothesis that TREM2 regulates Aβ deposition. The discovery that variants in *TREM2* strongly increase the odds of developing not only AD, but also Parkinson’s disease, amyotrophic lateral sclerosis, and frontotemporal dementia underscores the important role that the innate immune system plays in neurodegenerative disease and suggests that TREM2 subserves a beneficial microglial response in a variety of pathologies [36,37].

## Conclusions

Here, we report that loss of a single TREM2 allele decreases the number and size of plaque-associated microglia in 3-month old APPPS1-21 mice, but has no effect on total amyloid burden in either 3- or 7-month old APPPS1-21 mice. Individuals possessing a single variant TREM2 allele have substantially increased odds of developing AD, however, the role of TREM2 in AD pathology is unknown. To the best of our knowledge, this is the first report linking loss of a functional TREM2 allele to an observable phenotype in the presence of Aβ pathology.

## Methods

### Animals

APPPS1-21 transgenic mice (APP (KM670/671NL)/PS1 (L166P), gift of Mathias Jucker) were crossed with TREM2^−/−^ x CX3CR1^GFP/GFP^ mice or TREM2^+/+x^ CX3CR1^GFP/GFP^ mice to generate APPPS1-21 x TREM2^+/−^ CX3CR1^+/GFP^ (TREM2 Het) and APPPS1-21 x TREM2^+/+^ x CX3CR1^+/GFP^ (TREM2 WT) mice. All mice were maintained on a C57BL/6 background and all animal work was in accordance with guidelines established by the Animals Studies Committee at Washington University.

### Amyloid plaque analysis

Mice underwent transcardial perfusion with PBS (pH 7.4) followed by removal of the brain. Half the brain was fixed in 4% paraformaldehyde for 24 hours (4°C) and half was either frozen on dry ice and stored at −80°C for biochemical and qPCR analysis or processed to isolate microglial cells. Fixed hemibrains were cryoprotected in 30% sucrose in PBS (pH 7.4), frozen in dry ice, and serial coronal sections (50 μm thick) from the rostral anterior commissure to the caudal hippocampus were collected using a freezing sliding microtome. Three sections, 300 μm apart, were stained for Aβ using biotinylated HJ3.4 (anti-N-terminal Aβ antibody) and developed with DAB using a VECTASTAIN ABC Elite kit (Vector Labs) per manufacturer’s directions. To stain amyloid, three sections, 300 μm apart, were stained with X-34 dye (10 μM). HJ3.4 and X-34 stained sections were imaged using a NanoZoomer slide scanner (Hamamatsu Photonics) and the percent cortical area covered by HJ3.4 or X-34 staining was quantified by an experimenter blinded to the genotype and gender of the animal.

### Microglial isolation

A single cell suspension was generated from mouse hemibrains using a neural tissue dissociation kit (Miltenyi Biotec, 130-093-231) and gentleMACS Dissociator (Miltenyi Biotec) according to manufacturer recommended protocols. Microglia cells were then enriched by labeling the cells with mouse CD45 MicroBeads (MIltenyi Biotec, 130-052-301) and subsequent purification using a magnetic column. Microglia cells were then FACS sorted based on the surface markers of CD45^lo^, CD11b^high^ and GFP expression.

### Real-time qPCR analysis

RNA was extracted from frozen cortical tissue using the RNeasy kit (Qiagen) or from adult microglia using the RNeasy Micro kit (Qiagen). Reverse transcription was performed using a High-Capacity cDNA Reverse Transcription Kit (Life Technologies). Real-time qPCR was conducted with TaqMan primers (Life Technologies)[19] and the TaqMan Universal PCR Master Mix (Life Technologies) using an ABI Prizm 7500 thermocycler. Relative gene expression levels in TREM2 WT and TREM2 Het mice were compared using the ΔΔC_t_ method with β-actin used as a reference.

### Biochemical analysis of insoluble Aβ levels

Cortical tissue was sequentially homogenized in PBS (pH 7.4) and 5 M guanidine-Tris buffer (pH 8.0) in the presence of protease inhibitors (Roche). Aβ_40_ and Aβ_42_ levels were quantitatively measured by sandwich ELISA using either HJ2 (anti-Aβ_35–40_) or HJ7.4 (anti-Aβ_37–42_) as capture antibodies and biotinylated HJ5.1 (anti-Aβ_13–28_) as the detection antibody. Following incubation with poly-horseradish peroxidase-20 (Fitzgerald) ELISAs were developed using Super Slow ELISA TMB (Sigma).

### Microglia quantification

Alexa568-HJ3.4-stained brain sections were imaged using a 40x water-immersion objective (Zeiss, NA = 1.2) on a Zeiss LSM5 confocal microscope. All images were acquired and analyzed by an experimenter blinded to the genotype of the animal. Z-series stack images of randomly selected plaques within the lateral half of the cortex located above the hippocampus were then sequentially acquired for Alexa568 and GFP fluorescence (~12 optical sections, 3 μm apart). All images were acquired using identical acquisition parameters as 8-bit, 1024 × 1024 arrays. Z-series stacks were then converted to maximum intensity projections and threshold adjusted to isolate specific GFP fluorescence. Plaque-associated microglial coverage was assessed by measuring the percent area covered by GFP fluorescence within 20 μm of the edge of the plaque, including the area of the plaque itself. To assess the number and size of plaque-associated microglia, thresholded images were segmented using a watershed function and the number and area of microglia assessed in ImageJ using a minimum size cut-off of 16 μm^2^.

### Cytokine analysis

Cortical tissue from 3-month old APPPS1-21 x TREM2^+/+x^ CX3CR1^+/GFP^ and APPPS1-21 x TREM2^+/−^ CX3CR1^+/GFP^ was homogenized in 9x volumes lysis buffer (50 mM Tris–HCl (pH7.4), 2 mM EDTA, protease inhibitors). Lysates were centrifuged for 2 min at 13,000xg and analyzed using the Rodent Cytokine Multi-Analyte Profile (Myriad RBM).

### Statistics

Amyloid plaque immunohistochemistry and insoluble Aβ levels between male and female TREM2 WT and TREM2 Het mice were statistically analyzed using 2-way ANOVA (α = 0.05). The number, soma size, and percent-area covered by plaque-associated microglia were compared using a Mann Whitney test. RT-qPCR results from TREM2 WT and TREM2 Het groups were compared by t-test using a Benjamini-Hochberg correction for multiple comparisons. P-values less than 0.05 were considered statistically significant.

## Abbreviations

AD: Alzheimer’s disease; ADAD: Autosomal dominant Alzheimer’s disease; APP: Amyloid β (A4) precursor protein; PSEN1: Presenilin-1; PSEN2: Presenilin-2; LOAD: Late onset Alzheimer’s disease; apoE: Apolipoprotein E; TREM2: Triggering receptor expressed on myeloid cells-2; ITAM: Immunoreceptor tyrosine based activating motif; PLOSL: Polycystic lipomembranous osteodysplasia and sclerosing leukoencephalopathy; M-CSF: Macrophage colony stimulating factor; CSF1R: Colony stimulating factor 1 receptor.

## Competing interests

DMH co-founded and is on the scientific advisory board of C2N Diagnostics and currently serves as a consultant for Astra Zeneca, Eli Lilly, and Genentech.

## Authors’ contributions

Tissue immunohistochemistry and X-34 staining were performed by JDU, MBF, AS, TEM, FRS, and HJ. Aβ and X-34 staining were quantified by MBF and JDU. Microglial isolation was performed by YW and JDU. Tissue biochemistry was performed by MBF and JDU. Microglial localization was quantified by JDU. RT-qPCR was performed by JDU and AS. Experiments were conceived and designed by JDU, DMH, MC, and LP. Manuscript was written by JDU and critically reviewed by DMH, MC, and LP. All authors read and approved the final manuscript.
